# The Draft Genome of an Octocoral, *Dendronephthya gigantea*

**DOI:** 10.1093/gbe/evz043

**Published:** 2019-03-02

**Authors:** Yeonsu Jeon, Seung Gu Park, Nayun Lee, Jessica A Weber, Hui-Su Kim, Sung-Jin Hwang, Seonock Woo, Hak-Min Kim, Youngjune Bhak, Sungwon Jeon, Nayoung Lee, Yejin Jo, Asta Blazyte, Taewoo Ryu, Yun Sung Cho, Hyunho Kim, Jung-Hyun Lee, Hyung-Soon Yim, Jong Bhak, Seungshic Yum

**Affiliations:** 1 Korean Genomics Industrialization and Commercialization Center (KOGIC), Ulsan National Institute of Science and Technology (UNIST), Ulsan, Republic of Korea; 2 Department of Biomedical Engineering, School of Life Sciences, Ulsan National Institute of Science and Technology (UNIST), Ulsan, Republic of Korea; 3 Ecological Risk Research Division, Korea Institute of Ocean Science and Technology (KIOST), Geoje, Republic of Korea; 4 Department of Genetics, Harvard Medical School, Boston, Massachusetts; 5 Department of Biology, University of New Mexico; 6 Department of Life Science, Woosuk University, Republic of Korea; 7 Marine Biotechnology Research Center, Korea Institute of Ocean Science and Technology (KIOST), Busan, Republic of Korea; 8 APEC Climate Center, Busan, South Korea; 9 Clinomics Inc., Ulsan, Republic of Korea; 10 Personal Genomics Institute, Genome Research Foundation, Cheongju, Republic of Korea; 11 Faculty of Marine Environmental Science, University of Science and Technology (UST), Geoje, Republic of Korea

**Keywords:** soft coral, genome, octocoral, nonsymbiotic coral, cnidarian, *Dendronephthya gigantea*

## Abstract

Coral reefs composed of stony corals are threatened by global marine environmental changes. However, soft coral communities of octocorallian species, appear more resilient. The genomes of several cnidarians species have been published, including from stony corals, sea anemones, and hydra. To fill the phylogenetic gap for octocoral species of cnidarians, we sequenced the octocoral, *Dendronephthya gigantea*, a nonsymbiotic soft coral, commonly known as the carnation coral. The *D. gigantea* genome size is ∼276 Mb. A high-quality genome assembly was constructed from PacBio long reads (29.85 Gb with 108× coverage) and Illumina short paired-end reads (35.54 Gb with 128× coverage) resulting in the highest N50 value (1.4 Mb) reported thus far among cnidarian genomes. About 12% of the genome is repetitive elements and contained 28,879 predicted protein-coding genes. This gene set is composed of 94% complete BUSCO ortholog benchmark genes, which is the second highest value among the cnidarians, indicating high quality. Based on molecular phylogenetic analysis, octocoral and hexacoral divergence times were estimated at 544 MYA. There is a clear difference in *Hox* gene composition between these species: unlike hexacorals, the Antp superclass Evx gene was absent in *D. gigantea*. Here, we present the first genome assembly of a nonsymbiotic octocoral, *D. gigantea* to aid in the comparative genomic analysis of cnidarians, including stony and soft corals, both symbiotic and nonsymbiotic. The *D. gigantea* genome may also provide clues to mechanisms of differential coping between the soft and stony corals in response to scenarios of global warming.

## Introduction

Corals that belong to the phylum Cnidaria, class Anthozoa, provide habitats for a diversity of marine organisms (Friedlander and Parrish 1998) and are foundational members of the benthic community playing a major role in energy transfer between plankton and the benthos (van de Water et al. 2018). Corals capture large quantities of plankton and thereby regulate the primary and secondary production of the coastal food chains (Gili and Coma 1998; van de Water et al. 2018). Corals can be classified into hexacorals (stony corals and sea anemones) and octocorals (soft corals and sea fans). Global marine environmental changes, represented by seawater temperature rise and ocean acidification, are known to threaten coral reefs consisting of stony corals in tropical regions (Hoegh-Guldberg and research 1999; Carpenter et al. 2008). However, soft coral communities in temperate and subtropical regions, seem to prosper owing to their ability to disperse north as distribution limits extend (de Paula and Creed 2004; Santodomingo et al. 2013). To date much research has focused on understanding stony coral susceptibility to coral bleaching (Hoegh-Guldberg and research 1999) due to global warming and ocean acidification (Ries et al. 2010; de Putron et al. 2011; Pandolfi et al. 2011; Inoue et al. 2013). Yet, soft corals, which have sclerites, are less vulnerable to such environmental changes (Inoue et al. 2013) and it is suggested that temperate shallow-living octocorals are able to withstand increased levels of temperature and acidification (Lopes et al. 2018). Though, given the significant biological differences between the stony and soft corals in terms of calcification and survival strategies in the changing environment, only hexacoral genomes have been sequenced and analyzed (Putnam et al. 2007; Shinzato et al. 2011; Baumgarten et al. 2015; Snelling et al. 2017; Voolstra et al. 2017; Ying et al. 2018). Moreover, it is also beneficial to add an octocoral special to help understand the already available hexacoral genomes.

Here, we report the first genome assembly of an octocoral, *Dendronephthya gigantea*, commonly known as carnation coral. *D. gigantea* is a dominant species in the most southern coastal part of Korea (Hwang and Song 2007), in temperate and subtropical regions where yearly water temperature ranges from 14 °C to 26 °C (Hwang and Song 2007). In general, colonies of this species inhabit shallow water from 10 to 20 m in depth. It is an independent nonsymbiotic gonochoric internal brooder. It preys on zooplankton and phytoplankton and does not possess zooxanthellae (Imbs et al. 2007) in contrast to reef-building *Acropora* species. Our draft genome may therefore serve as a resource for evolutionary studies of azooxanthellate octocorals in terms of understanding different coping strategies mediating against rapid environmental changes in comparison to published stony coral genomes.

### Sequencing and De Novo Genome Assembly

We estimated the genome size of *D. gigantea* to be 276 Mb (276,273,039 bp) using Illumina HiSeq 2500 short paired-end reads (35.54 Gb with 128-fold coverage) of at a k-mer size of 17. The graph for the k-mer frequency distribution showed that there were two peaks and the heterozygosity of the *D. gigantea* genome is high (Liu et al. 2013) (supplementary fig. 1, Supplementary Material online). This finding is consistent with previous reports of invertebrates showing relatively high levels of genome heterozygosity (Ellegren and Galtier 2016).

We used PacBio long reads (29.85 Gb with 108-fold coverage) for an initial draft assembly which is complemented by Illumina short paired-end reads (35.54 Gb with 128-fold coverage) for error-correction. Bacterial and fungal DNA reads (1.18%) were filtered out during genome assembly. The final assembly resulted in a 286 Mb genome, which covers 103.58% of the estimated genome size of 276 Mb (Table 1). The final contig N50 value achieved was 1,445,523 bp (Table 1). The *D. gigantea* genome assembly produced has the longest N50 length (1.4 Mb) reported among cnidarian genomes thus far (Table 1). In addition, the self-mapping rate of Illumina short paired-end reads to the genome assembly was very high (95.9%).

**Table 1:** Statistics of the *Dendronephthya gigantea* Genome Assembly Compared to Other Cnidarians

	*Dendronephthya gigantea*	*Orbicella faveolata*	*Stylophora pistillata*	*Acropora digitifera*	*Aiptasia pallida*	*Nematostella vectensis*	*Hydra magnipapillata*
No. of sequences	1,323	1,933	5,688	2,421	4,312	10,804	20,916
Total bases (bp)	286,131,912	485,548,939	400,120,318	447,497,157	256,132,296	356,613,585	852,170,992
Average length (bp)	216,275	251,189	70,345	184,839	59,400	33,008	40,743
SD (bp)	596,503	541,789	193,436	280,650	169,768	149,438	58,784
N50 (bp)	1,445,523	1,162,446	457,453	483,559	442,145	472,588	96,317
GC contents	37%	39%	39%	39%	36%	41%	28%

### Gene Prediction, Annotation, and Quality Assessment

We found close to 29,000 protein-coding genes in *D. gigantea* using two different methodologies (see supplementary table 1, Supplementary Material online). The first and second approach predicted 28,879 and 28,937 protein-coding genes in the *D. gigantea* genome, respectively.

We compared both gene sets using BUSCO (version 3.0.2) (Simão et al. 2015; Waterhouse et al. 2018) which showed comparable high quality, increasing our confidence in the predicted gene set. The gene set obtained by the first method showed a slightly higher quality (93.97% complete BUSCO genes) than that of the second method (93.35%) (supplementary table 2, Supplementary Material online).

The *D. gigantea* gene set was of high quality among the cnidarians and covered ∼94% of the complete BUSCO ortholog benchmark genes (supplementary fig. 2, Supplementary Material online). We compared the quality of the *D. gigantea* gene models with six published cnidarians (*Aiptasia pallida*, *Acropora digitifera*, *Hydra magnipapillata*, *Nematostella vectensis*, *Orbicella faveolata*, and *Stylophora pistillata*). The *D. gigantea* gene models had ∼87% complete single copy BUSCO genes (supplementary fig. 2, Supplementary Material online). It also had the second highest value of complete BUSCO genes (∼94%) which included both single copy and duplicated genes among cnidarians (supplementary fig. 2, Supplementary Material online).

Almost 12% of the *D. gigantea* genome consists of repeat elements. We found transposable elements make up an 11.97% of the *D. gigantea* genome, in which tandem repeats and long terminal repeat elements (LTR) represented 7.24% and 2.25% of the genome, respectively (supplementary table 3, Supplementary Material online).

### Phylogenetic Analysis and *Hox* Gene Clusters Identification

**Fig. 1.**
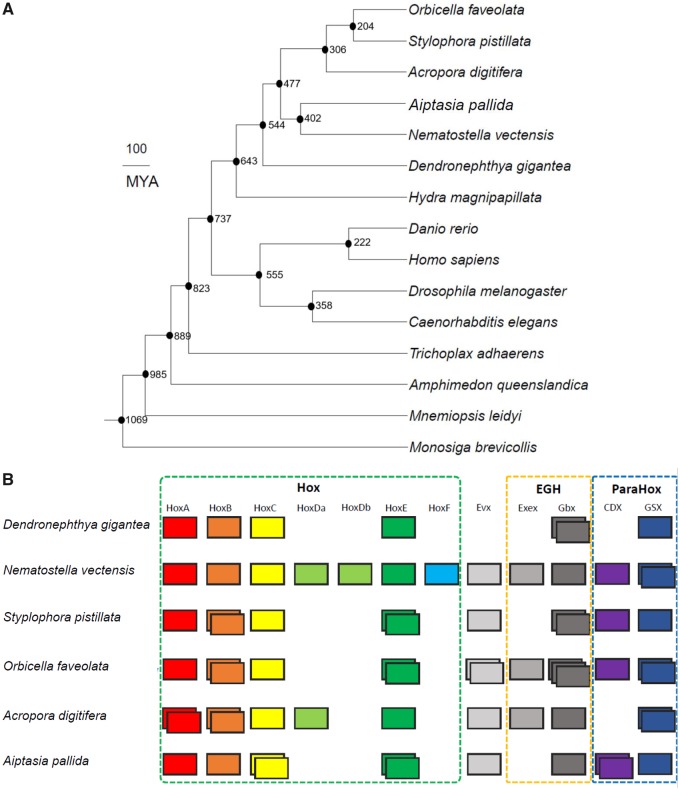
—Phylogenetic relationship and *Hox* gene clusters of *Dendronephthya gigantea* and other species. (*A*) Tree shows the phylogeny with divergence time among 15 species. Numbers in each branch denote the estimated divergence time (MYA). (*B*) Green dashed-line box denotes *Hox* gene cluster (*HoxA*, *Hox*B, *Hox*C, *Hox*Da, *Hox*Db, *Hox*E, and *HoxF*), yellow dashed-line box denotes *EGF* gene cluster (*Evex* and *Gbx*), and blue dashed-line box denotes Para*Hox* gene cluster (*CDX* and *GSX*). The number of boxes shows the number of each gene copies in the genome.

We found that *D. gigantea* has diverged the earliest among the anthozoans based on our calculations. We identified that *D. gigantea* contains 12,597 orthologous gene families, excluding singletons, and 3,656 of them are shared with stony corals (*Orbicella faveolata*, *Stylophora pistillata*, and *Acropora digitifera*) and hydra (*Hydra magnipapillata*) (supplementary fig. 3, Supplementary Material online). A total of 4,863 gene families were *D. gigantea*-specific (supplementary fig. 3, Supplementary Material online). Second, we use molecular phylogenetic analysis to show that the octocoral, *D. gigantea*, is positioned between hexacorallia and hydrozoa (fig. 1*A*), implying that the octocoral is the earliest diverged group among anthozoans. Divergence time estimation analysis suggested the divergence of the octocoral (*D. gigantea*) from the other three stony corals (*O. faveolata*, *S. pistillata*, and *A. digitifera*) happened 544 MYA (fig. 1*A*).

We also examined the differences of *Hox* (Homeobox) genes between the soft and stony corals. *Hox* genes encode transcription factors that perform diverse roles during development (Akam 1995). They are best known to define body plan (Akam 1995). We found the three stony corals have a similar and familiar pattern of *Hox* gene clusters (Ying et al. 2018) (fig. 1*B*). However, *Evx*, which is a member of the Antp superclass of *Hox* genes (Patel and Prince 2000), is absent in *D. gigantea* (fig. 1*B*) a finding that should be verified experimentally.

Here, we present a high-quality, draft genome of *Dendronephthya gigantea*, the first nonsymbiotic octocoral. Our analyses show the octocoral is the earliest diverged group among anthozoans with an estimated divergence time of 544 MYA from the hexacorals. It adds a new octocoral assembly for cnidarians, in addition to hexacoral and hydra genomes, thus it facilitates in depth comparative analyses of stony and soft corals that are either symbiotic and/or nonsymbiotic. The *D. gigantea* genome will support future experiments aimed at determining differences in the genetic coping mechanisms between soft and stony corals in terms of calcification and survival strategies in the face of global warming and ocean acidification.

## Materials and Methods

### Genome Assembly and Annotation

A detailed description of the sample collection, DNA extraction, RNA extraction, genome size estimation, de novo genome assembly, and genome annotation can be found in the Supplementary Material online. In brief, PacBio long reads were used for a draft assembly processed by FALCON (version 0.3.0) (Chin et al. 2016) complemented by Illumina short paired-end reads for error-correction. We mapped Illumina short paired-end reads to the genome assembly to confirm the high quality using BWA (version 0.7.12) (Li,2013) resulting in a 95.9% mapping rate. For gene prediction, we merged ab initio- and homology-based predictions using AUGUSTUS (version 3.1) (Stanke et al. 2008) with additional information obtained from homology-based predicted *D. gigantea* gene models, RNA-seq data of the planula and polyp of *D. gigantea* and polyps of *Scleronephthya gracillimum* (unpublished data), and Expressed Sequence Tags (ESTs) of corals downloaded from NCBI database.

### Phylogenetic Analysis and *Hox* Gene Clusters Identification

We examined orthologous gene clustering of complete protein-coding genes from the six published cnidarians (*Orbicella faveolata*, *Stylophora pistillata*, *Acropora digitifera*, *Nematostella vectensis*, *Aiptasia pallida*, and *Hydra magnipapillata*) and seven noncnidarian metazoans (*Danio rerio*, *Homo Sapiens*, *Drosophila melanogaster*, *Caenorhabditis elegans*, *Trichoplax adhaerens*, *Amphimedon queenslandica*, and *Mnemiopsis leidyi*). Our outgroup was the unicellular holozoan, *Monosiga brevicollis*. Clusters were generated using OrthoMCL (version 2.0.9) (Li et al. 2003) with an E-value cutoff of 1E-20.

We estimated the phylogeny using 197 single copy orthologs using the PROTGAMMAJTT model in RAxML (version 8.2.8) (Stamatakis 2014). The divergence times were estimated using the MCMCtree program in PAML package (version 4.8) (Yang 2007) with the independent rates model (clock = 2). The date of the node between *D. melanogaster*–*C. elegans* was constrained to 743 MYA and *H. sapiens*–*D. rerio* was constrained to 435 MYA based on the TimeTree database (Kumar et al. 2017).

To identify and classify *Hox* gene cluster patterns, we sought for all instances of the homeobox domain based on Pfam database (Finn et al. 2016) using HMMER (version 3.1b2) (Finn et al. 2011) and InterProScan (version 5.32-71.0) (Jones et al. 2014; Mitchell et al. 2018). Homeobox domain genes were classified using BLAST (version 2.2.28) (Altschul et al. 1990) against HomeoDB (Zhong et al. 2008; Zhong and Holland 2011) and mapping to the homeobox domain of *N. vectensisHox* genes from GenBank (Lipman, et al. 2016). 

## Supplementary Material

Supplementary data are available at *Genome Biology and Evolution* online.

## Supplementary Material

Supplementary Data

## Notes

**Data deposition:** The octocoral whole genome and transcriptome project has been deposited at DDBJ/ENA/GenBank under the accession PRJNA507923 and PRJNA507943. DNA and RNA sequencing reads have been uploaded to the NCBI Read Archive under the accession (SRR8293699, and SRR8293698 and SRR8293935, and SRR8293936), respectively. The genome assembly has been deposited at DDBJ/ENA/GenBank under the accession RSEI01000000.
