# Supplementary material for: The Draft Genome of an Octocoral, Dendronephthya gigantea
Source: Genome Biol Evol. 2019 Mar 2;11(3):949–53. doi: 10.1093/gbe/evz043 (PMC6447388; doi:10.1093/gbe/evz043)
Supplement: Supplementary Data [file evz043_supp.zip › Supplementary_figure3.pdf]

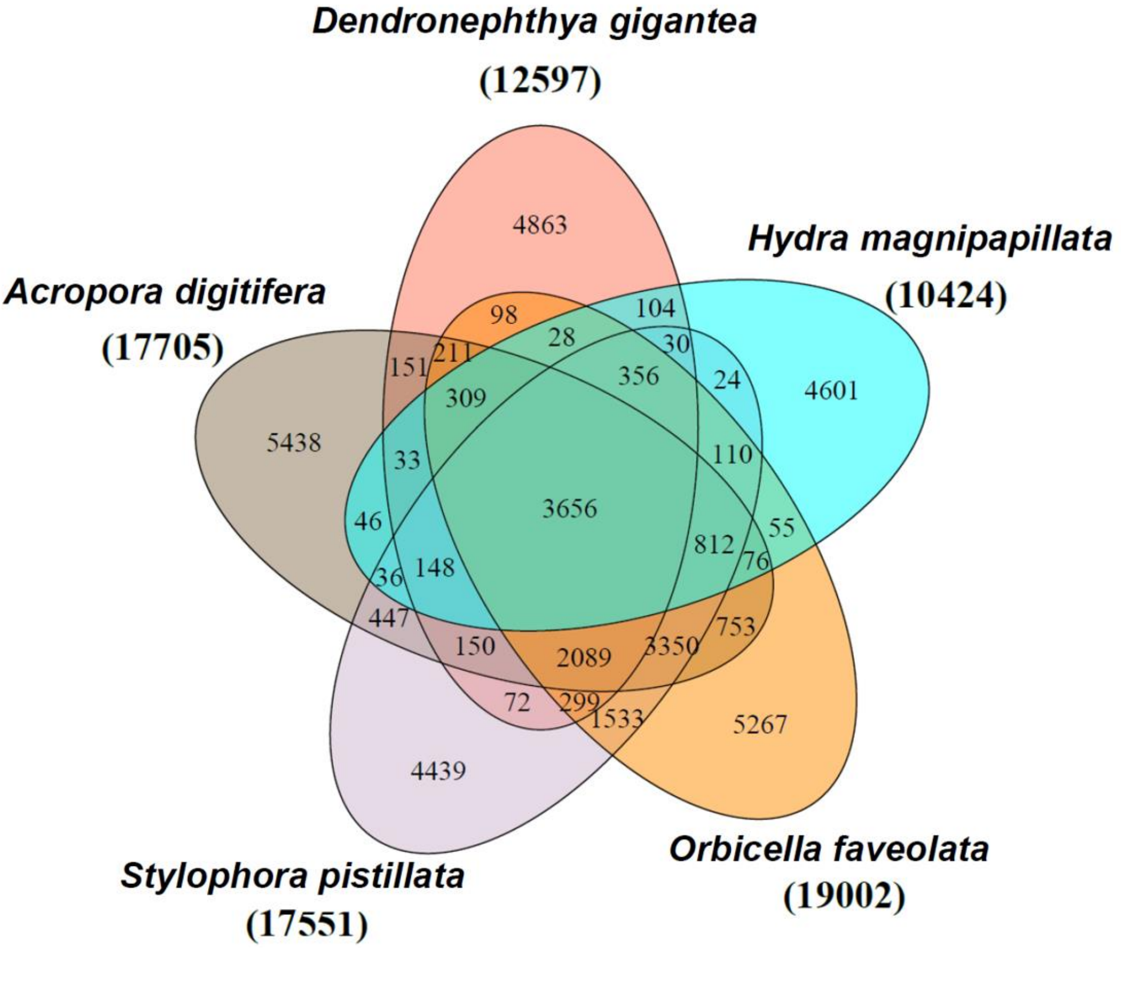
Dendronephthya gigantea
(12597)
Hydra magnipapillata
(10424)
Acropora digitifera
(17705)
Stylophora pistillata
(17551)
Orbicella faveolata
(19002)
4863
98
104
28
30
151
211
356
24
309
4601
5438
33
110
3656
46
55
812
76
148
36
447
753
150
2089
3350
72
299
1533
5267
4439
