# Supplementary material for: The Draft Genome of an Octocoral, Dendronephthya gigantea
Source: Genome Biol Evol. 2019 Mar 2;11(3):949–53. doi: 10.1093/gbe/evz043 (PMC6447388; doi:10.1093/gbe/evz043)
Supplement: Supplementary Data [file evz043_supp.zip › Supplementary_materials_final.docx]

**Supplementary materials**

**1. Sample collection**

A *D. gigantea* colony was collected at approximately 20 m underwater near Seogwipo, Jeju Island, South Korea (33° 13′39″ N, 126° 34′03″ E) on May 22, 2015 using standard scuba techniques. The underwater yearly temperature range of the site was measured to range between 15 and 26 °C. A colony of *D. gigantea,* which carries mature oocytes in the gastrodermal canals, was transported to the laboratory on August 20, 2016 for observation of planula development. After planulation the development of an early planula into a primary polyp was observed under a stereomicroscope and samples for RNA-seq were acquired.

**2. DNA extraction**

For the DNA extraction, the *D. gigantea* colony was mortar-pulverized in liquid nitrogen and the powder homogenized in lysis solution [2% CTAB, 1.4M NaCl, 100 mM Tris-Cl (pH 8.0), 20 mM EDTA, 1% β-mercaptoethanol], and incubated at 65°C for one hour. The same volume of a phenol:chloroform:isoamyl alcohol (23:24:1) mixture was added to denature the proteins and followed by phase separation by centrifugation at 12,000 rpm for 15 min at room temperature. The aqueous phase was retained and incubated at 37°C for one hour after RNase A (30 mg/ml) was added. The DNA was extracted with a phenol:chloroform:isoamyl alcohol (25:24:1) mixture treatment, followed by adding a chloroform:isoamyl alcohol (24:1) mixture with separating phases centrifuged at 10,000g for 15 min at room temperature. In the next step 1/10 volume of 3 M sodium acetate (pH 5.2) and the same volume of 100% ethanol were added into the retained aqueous phase. The precipitated DNA was washed using 70% ethanol and re-suspended in an appropriate volume of ion-exchanged ultrapure water. The DNA quantity was verified by picogreen method using Victor 3 fluorometry and agarose gel electrophoresis.

**3. RNA extraction**

To extract RNA, the *D. gigantea* whole colony and planula larvae were mortar-pulverized in liquid nitrogen. The tissue powder was then homogenized in 700 µl of lysis solution [35 mM EDTA, 0.7 M LiCl, 7% SDS, 200 mM Tris-Cl (pH 9.0)], and RNA was extracted with 700 µl of water-saturated phenol. A one-third of volume of 8 M LiCl was added into the retained aqueous phase, which was incubated at 4°C for two hours. The RNA was precipitated after centrifugation at 14,000 rpm for 30 min followed by resuspension in 300 µl of DEPC-treated water followed by a reprecipitation with 1/10 volumes of 3 M sodium acetate (pH 5.2) and isopropanol. The precipitated RNA was rinsed with 70% ethanol (diluted in DEPC-treated water) and dissolved in an appropriate volume of DEPC-treated water (30–40 µl). RNA quantity and integrity were analyzed using a NanoDrop ND-1000 spectrometer and an Agilent 2100 Bioanalyzer with RNA Integrity Number (RIN).

**4. Genome size estimation and *de novo* genome assembly**

A short-read DNA sequencing and k-mer analysis were performed to estimate the genome size using an Illumina HiSeq 2500 platform and SOAPec (version 2.01) (Luo, et al. 2012).

For a *de novo* genome assembly, PacBio RS II long reads and Illumina HiSeq 2500 short reads data were used. The PacBio long reads were used for an initial draft assembly processed by FALCON (version 0.3.0) (Chin, et al. 2016) and was complemented by the Illumina short paired-end reads for error-correction. We filtered out bacterial and fungal DNA reads using BLASTN (version 2.2.28) (Altschul, et al. 1990) against the UniProt database (research 2018). For error-correction, we replaced the assembled contigs of PacBio long-reads with the Illumina short paired-end reads by self-mapping in case of homo-variants and non-reference hetero variants. We repeated this error-correction process three times to correct for sequencing errors.

**5. Annotation of repetitive sequences**

We searched for transposable elements using both *ab initio*- and homology-based methods using RepeatModeler (version 1.0.7) (Price, et al. 2005) and RepeatMasker (version 4.0.5) (Chen 2004) and RepeatMasker (version 4.0.5) (Chen 2004) and Repbase database (version 19.03) (Jurka, et al. 2005), respectively. Tandem repeat predictions were performed using Tandem Repeats Finder (version 4.07) (Benson 1999). The results were merged together.

**6. Genome annotation**

We selected our final gene set after comparing two methods. First, we merged *ab initio*- and homology-based predictions using AUGUSTUS (version 3.1) (Stanke, et al. 2008) with additional information obtained from homology-based predicted *D. gigantea* gene models, RNA-seq data of the planula and polyp of *D. gigantea* and polyps of *Scleronephthya gracillimum* (unpublished data), and Expressed Sequence Tags (ESTs) of corals downloaded from NCBI database (Benson, et al. 2017). We used homology-based methods to align repeat-masked *D. gigantea* genome to proteomes of cnidarians obtained from the UniProt database (research 2018), *H. sapiens*, *M. musculus*, and *D. rerio* using GenBlastA (version 1.0.4) (She, et al. 2009) with E-value cutoff 1E-05 and Exonerate (version 2.2.0) (Slater and Birney 2005). We gained 8,669 gene models from the homology-based method and these were used as exon hints when we merged both of the *ab initio*- and homology-based methods. In addition, we aligned RNA-seq reads to the *D. gigantea* genome using TopHat (version 2.0.9) (Trapnell, et al. 2009) to use as intron hints. The EST sequences of corals were mapped to the genome assembly using BLAT (version 34) (Kent 2002) and used as exon and intron hints. The gene models were filtered according to these criteria: final gene models must contain both start and stop codons, CDS length is a multiple of three, and the length of protein-coding genes is more than 40 amino acids. In addition, single exon genes with FPKM value < 1 were filtered out when multiple exons existed with the same gene symbol from the UniProt database (research 2018).

In a second approach, we combined predicted genes from the Maker pipeline (version 2.31.10) (Cantarel, et al. 2008) with those from BRAKER2 (version 2.1.2) (Hoff, et al. 2015; Stanke, et al. 2008; Stanke, et al. 2006). To obtain additional evidence for predicted genes, we mapped assembled transcripts from *D. gigantea* planula and polyp RNA-seq data, sequences from Swiss-Prot database (research 2018), and genes from closely related species to the *D. gigantea* genome using BLAST (version 2.2.28) (Altschul, et al. 1990) and Exonerate (version 2.2.0) (Slater and Birney 2005). Predicted genes with less than 1.00 AED scores were sorted as the final set. The best gene models were selected by comparing the genes from the BRAKER2 (Hoff, et al. 2015; Stanke, et al. 2008; Stanke, et al. 2006) with those from the Maker pipeline (Cantarel, et al. 2008).

The final gene set was the one with a better BUSCO results from the two approaches (version 3.0.2) (Simão, et al. 2015; Waterhouse, et al. 2017).

**Supplementary Tables**

**Supplementary Table 1: Statistics of protein-coding genes in *D. gigantea***

|  | Number | Percentage (%) |
| --- | --- | --- |
| Pre-filtered gene models | 32,487 | 100.00 |
| Gene models with amino acid length > 40 | 32,478 | 99.97 |
| Gene models whose CDS length is multiple of 3 | 32,256 | 99.29 |
| Complete gene models containing both start and stop codons | 32,150 | 98.96 |
| Single exon genes with FPKM value < 1 when multi exon gene exist as same symbol | 3,310 | 10.19 |
| Total number of final gene models | 28,879 | 88.89 |

**Supplementary Table 2: Comparison of BUSCO assessments of the gene sets between two methods**

|  | Current gene set | | | Not current gene set | |
| --- | --- | --- | --- | --- | --- |
|  | | First method | | Second method | |
|  | | Number | Percentage (%) | Number | Percentage (%) |
| Complete single copy BUSCO genes | | 854 | 87.32 | 806 | 82.41 |
| Complete duplicated BUSCO genes | | 65 | 6.65 | 107 | 10.94 |
| Complete BUSCO genes (single copy + duplicated) | | 919 | 93.97 | 913 | 93.35 |
| Fragmented BUSCO genes | | 24 | 2.45 | 35 | 3.58 |
| Missing BUSCO genes | | 35 | 3.58 | 30 | 3.07 |
| Total of used genes in BUSCO | | 978 | - | 978 | - |

**Supplementary Table 3: Repeat sequences in the *D. gigantea* genome**

| Repeat type | Ab initio based (bp) | Homology based (bp) | Total (bp) | Percentage of genome (%) |
| --- | --- | --- | --- | --- |
| DNA | 5,989,055 | 2,444,506 | 6,344,179 | 2.22 |
| LINE | 2,621,991 | 1,893,795 | 3,014,162 | 1.05 |
| LTR | 6,186,765 | 4,707,866 | 6,435,444 | 2.25 |
| Low complexity | 36,863 | 41,827 | 42,373 | 0.015 |
| SINE | 4,753 | - | 4,753 | 0.0017 |
| Satellite | 244,013 | 9,371 | 244,167 | 0.085 |
| Simple repeat | 619,655 | 1,721,644 | 1,727,993 | 0.60 |
| Tandem repeat^†^ | - | - | 20,729,359 | 7.24 |
| Unknown | 142,961 | 242,937 | 253,480 | 0.09 |
| Unspecified | 2,153,035 | - | 2,153,035 | 0.75 |
| Total transposable elements | 16,760,059 | 10,828,627 | 34,254,188 | 11.97^‡^ |

^†^ Tandem repeats were separately predicted.

^‡^ The total element sum is smaller than the arithmetic sum of the repeat types because there are overlapped repeats.

**Supplementary figure legends**

**Supplementary Figure 1. K-mer (17-mer) frequency percentage distribution curve of *D. gigantea* sequencing reads.**

The X-axis represents the k-mer depth (x) and the Y-axis represents the percentage of specific k-mer. There are two peaks in the graph, implying the heterozygosity of the *D. gigantea* genome is high. The left and right peak appear when the k-mer depth is 56 and 113, respectively. The genome size was estimated to be 276 Mb.

**Supplementary Figure 2. Assessment of the *D. gigantea* gene models compared to other cnidarians.**

The figure shows results of BUSCO analysis. Light-blue denotes the complete single-copy genes, dark-blue denotes complete duplicated genes, yellow denotes fragmented genes, and red denotes missing genes.

**Supplementary Figure 3. A Venn-diagram of orthologous gene families.**

The Venn-diagram shows shared and specific gene families in the *D. gigantea*, *A. digitifera*, *S. pistillata*, *O. faveolata*, and *H. magnipapillata* genomes. The total numbers of gene families are given in parentheses.

**References**

Altschul SF, Gish W, Miller W, Myers EW, Lipman DJJJomb 1990. Basic local alignment search tool. 215: 403-410.

Benson DA, et al. 2017. GenBank. Nucleic acids research.

Benson GJNar 1999. Tandem repeats finder: a program to analyze DNA sequences. 27: 573-580.

Cantarel BL, et al. 2008. MAKER: an easy-to-use annotation pipeline designed for emerging model organism genomes. Genome research 18: 188-196.

Chen NJCpib 2004. Using RepeatMasker to identify repetitive elements in genomic sequences. 5: 4.10. 11-14.10. 14.

Chin C-S, et al. 2016. Phased diploid genome assembly with single-molecule real-time sequencing. 13: 1050.

Hoff KJ, Lange S, Lomsadze A, Borodovsky M, Stanke M 2015. BRAKER1: unsupervised RNA-Seq-based genome annotation with GeneMark-ET and AUGUSTUS. Bioinformatics 32: 767-769.

Jurka J, et al. 2005. Repbase Update, a database of eukaryotic repetitive elements. 110: 462-467.

Kent WJ 2002. BLAT—the BLAST-like alignment tool. Genome research 12: 656-664.

Luo R, et al. 2012. SOAPdenovo2: an empirically improved memory-efficient short-read de novo assembler. 1: 18.

Price AL, Jones NC, Pevzner PAJB 2005. De novo identification of repeat families in large genomes. 21: i351-i358.

research UCJNa 2018. UniProt: the universal protein knowledgebase. 46: 2699.

She R, Chu JS-C, Wang K, Pei J, Chen N 2009. GenBlastA: enabling BLAST to identify homologous gene sequences. Genome research 19: 143-149.

Simão FA, Waterhouse RM, Ioannidis P, Kriventseva EV, Zdobnov EM 2015. BUSCO: assessing genome assembly and annotation completeness with single-copy orthologs. Bioinformatics 31: 3210-3212.

Slater GSC, Birney E 2005. Automated generation of heuristics for biological sequence comparison. BMC bioinformatics 6: 31.

Stanke M, Diekhans M, Baertsch R, Haussler D 2008. Using native and syntenically mapped cDNA alignments to improve de novo gene finding. Bioinformatics 24: 637-644.

Stanke M, Schöffmann O, Morgenstern B, Waack S 2006. Gene prediction in eukaryotes with a generalized hidden Markov model that uses hints from external sources. BMC bioinformatics 7: 62.

Trapnell C, Pachter L, Salzberg SL 2009. TopHat: discovering splice junctions with RNA-Seq. Bioinformatics 25: 1105-1111.

Waterhouse RM, et al. 2017. BUSCO applications from quality assessments to gene prediction and phylogenomics. Molecular biology and evolution 35: 543-548.
