# Supplementary figures and images for: The Draft Genome of an Octocoral, Dendronephthya gigantea
Source: Genome Biol Evol. 2019 Mar 2;11(3):949–53. doi: 10.1093/gbe/evz043 (PMC6447388; doi:10.1093/gbe/evz043)

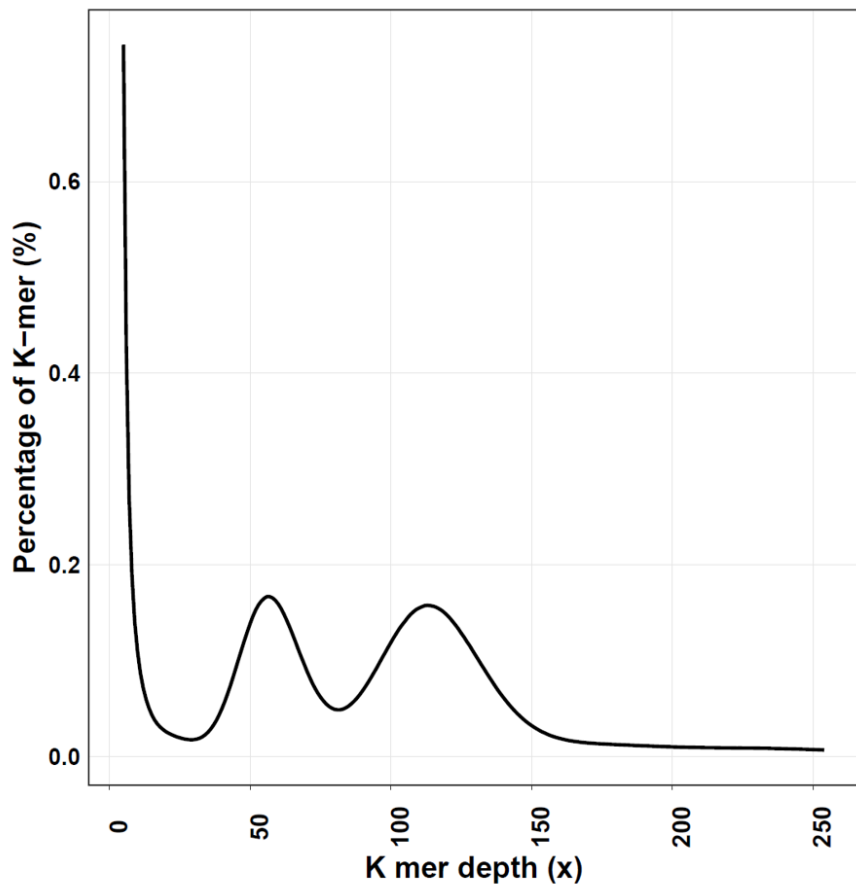

Supplement: Supplementary Data [file evz043_supp.zip › Supplementary_figure1.pdf]

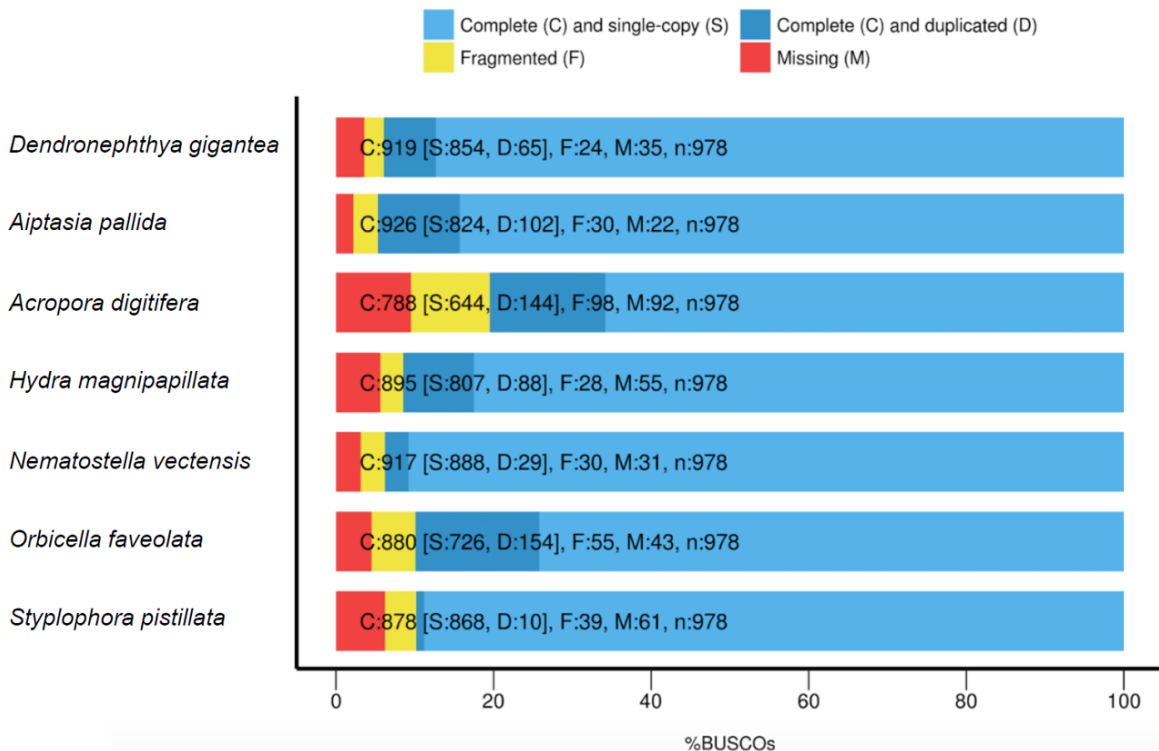

Supplement: Supplementary Data [file evz043_supp.zip › Supplementary_figure2.pdf]
